# EvoDB: a database of evolutionary rate profiles, associated protein domains and phylogenetic trees for PFAM-A

**DOI:** 10.1093/database/bav065

**Published:** 2015-07-02

**Authors:** Andrew Ndhlovu, Pierre M. Durand, Scott Hazelhurst

**Affiliations:** ^1^Evolutionary Medicine Laboratory, Department of Molecular Medicine and Haematology, Faculty of Health Sciences,; ^2^Sydney Brenner Institute of Molecular Bioscience, The Mount, 9 Jubilee Road, Parktown 2193, Johannesburg, South Africa,; ^3^Department of Biodiversity and Conservation Biology, Faculty of Natural Sciences, University of the Western Cape, Private Bag X17, Belville, Cape Town 7530, South Africa,; ^4^Department of Ecology and Evolutionary Biology, University of Arizona, Tucson, AZ 85721, USA and; ^5^School of Electrical and Information Engineering, University of the Witwatersrand, The Mount, 9 Jubilee Road, Parktown 2193, Johannesburg, South Africa

## Abstract

The evolutionary rate at codon sites across protein-coding nucleotide sequences represents a valuable tier of information for aligning sequences, inferring homology and constructing phylogenetic profiles. However, a comprehensive resource for cataloguing the evolutionary rate at codon sites and their corresponding nucleotide and protein domain sequence alignments has not been developed. To address this gap in knowledge, EvoDB (an Evolutionary rates DataBase) was compiled. Nucleotide sequences and their corresponding protein domain data including the associated seed alignments from the PFAM-A (protein family) database were used to estimate evolutionary rate (ω = d*N*/d*S*) profiles at codon sites for each entry. EvoDB contains 98.83% of the gapped nucleotide sequence alignments and 97.1% of the evolutionary rate profiles for the corresponding information in PFAM-A. As the identification of codon sites under positive selection and their position in a sequence profile is usually the most sought after information for molecular evolutionary biologists, evolutionary rate profiles were determined under the M2a model using the CODEML algorithm in the PAML (Phylogenetic Analysis by Maximum Likelihood) suite of software. Validation of nucleotide sequences against amino acid data was implemented to ensure high data quality. EvoDB is a catalogue of the evolutionary rate profiles and provides the corresponding phylogenetic trees, PFAM-A alignments and annotated accession identifier data. In addition, the database can be explored and queried using known evolutionary rate profiles to identify domains under similar evolutionary constraints and pressures. EvoDB is a resource for evolutionary, phylogenetic studies and presents a tier of information untapped by current databases.

**Database URL:** http://www.bioinf.wits.ac.za/software/fire/evodb

## Introduction

Hypothesis testing in molecular evolution and phylogenetics depends upon accurate sequence alignments. These data can then be used to investigate adaptation at the sequence level by, for example, detecting protein domains, individual codon sites or branches under positive selection (1); or developing resources for molecular evolutionary studies (2). However, the availability of protein domains and their corresponding nucleotide sequences linked to estimates of sequence evolutionary rates is lacking. Furthermore, a comprehensive database of sequence evolutionary rate profiles, which could be probed with a query sequence of a known evolutionary rate profile, is currently unavailable.

To address this gap, EvoDB (an Evolutionary rates DataBase) was compiled. The CODEML program in the PAML (Phylogenetic Analysis using Maximum Likelihood) suite of software (ver. 4.4) (3) was utilized for estimating evolutionary rates (by convention designated as ω = d*N*/d*S*) although any similar methodology (e.g. HyPhy) can be used (4). Evolutionary rates can be determined for whole sequences (the entire protein or protein domain), lineages within a phylogenetic tree or at particular codon sites. Typically, molecular evolutionary biologists are interested in identifying specific codon sites under positive selection or adaptive evolution (5) or in the pattern of evolutionary rates at codon sites across a sequence (6). In the PAML suite of software the model (NSsites) M2a, which uses nucleotide coding sequence data to calculate the evolutionary rate at codon sites, was therefore used in the development of this database and for explanatory purposes although any of the alternate models can be substituted. In addition to the M2a model, M1a analyses are also provided. The PFAM-A seed alignments (7) provided a suitable framework for establishing a database that comprised a catalogue of the evolutionary rate at codon sites for each protein domain entry. The PANDIT (Protein and Associated Nucleotide Domains with Inferred Trees) database is a compilation of nucleic acid sequences and corresponding phylogenetic trees for the PFAM-A database; however, this database has not been updated since version 17.0 of PFAM in 2005 (8). Nucleotide sequence data for each domain in the PFAM-A database are obtained by cross reference mapping information in the PFAM Stockholm file. This information is cross-referenced to a Swiss-Prot (9) protein which provides the accession identifier and feature information for the corresponding GenBank (10) file.

EvoDB comprises protein domains from PFAM-A, the corresponding nucleotide sequences and estimates of their evolutionary rates based upon the PFAM-A (ver. 27.0) seed alignments. The database is a compilation of evolutionary rates linked to amino acid and nucleotide data and can be queried using evolutionary rate estimates (under model M2a). The conceptualization and implementation of such an approach have been described elsewhere (11) and a newer version is forthcoming (manuscript in review).

## Methods

### Implementation

**Figure 1.**
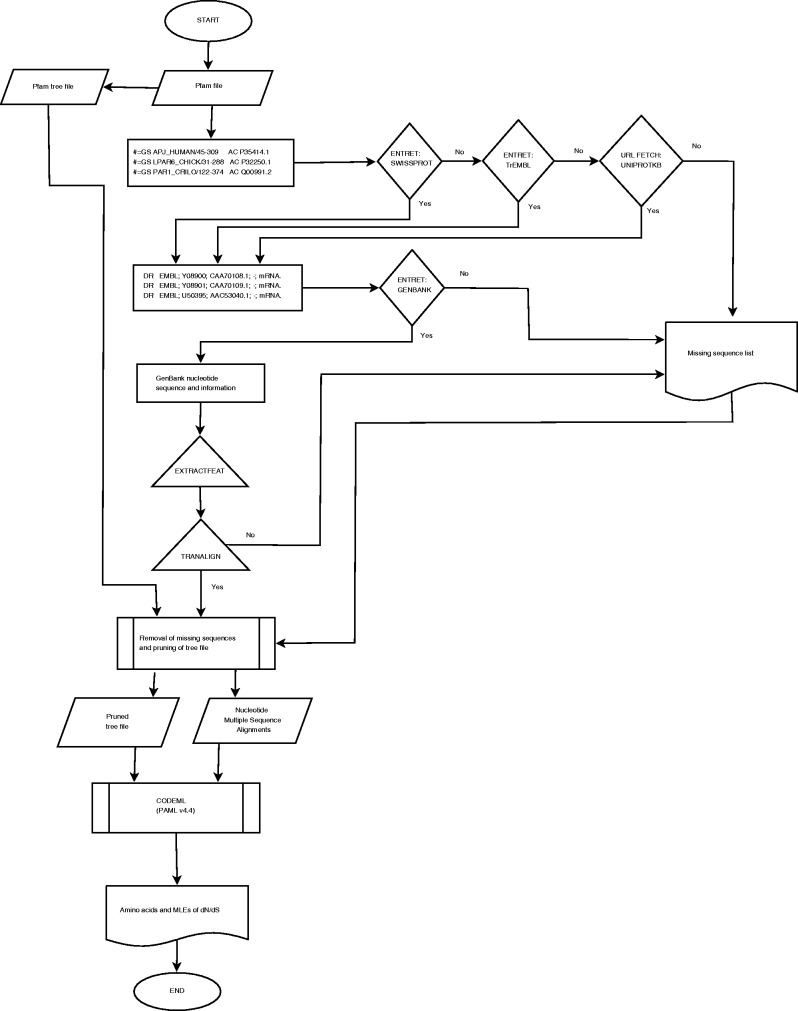
Workflow for the development and compilation of EvoDB.

The retrieval of sequences and the determination of the ω maximum-likelihood estimate (MLE) profiles using CODEML are computationally expensive. The computational resources of the Wits Core Cluster (ZA-WITS-CORE) of 13 nodes running Scientific Linux 6.3 were utilized for achieving parallelism. PFAM-A seed alignments and the GenBank database, including the Swiss-Prot and TrEMBL in the Uniprot resource (9) database were made available locally. Resources and scheduling were managed by Maui/PBS and TORQUE systems, respectively. Each PFAM entry was submitted as a job using custom scripts. The compilation pipeline is provided in Figure 1.

### Sequence retrieval, validation and determination of ω MLEs profiles

Accession identifiers for protein sequences were extracted from PFAM Stockholm files using the generic per-sequence annotation ‘#=GS AC’ tag. These identifiers were used to retrieve protein sequences from the Swiss-Prot database using the ENTRET program in the EMBOSS (European Molecular Biology Open Software Suite) software package (ver. 6.3.1) (12). Those sequences that could not be found on Swiss-Prot were queried in the computer curated TrEMBL database; otherwise, they were removed from the alignments. Each Swiss-Prot protein sequence file contains mapping accession numbers for the corresponding nucleotide sequences found in the ‘DR EMBL’ database cross-reference annotation. Accession and feature information from the Swiss-Prot files was used to retrieve the corresponding nucleotide files from the GenBank database (ver. 193.0) using the ENTRET program. The accession identifier and feature information were used to retrieve the coding sequences (CDS) from GenBank files using the EXTRACTFEAT program in EMBOSS.

The TRANALIGN program in EMBOSS finds corresponding nucleotide CDS for a protein sequence by comparing the translated nucleotide to the protein sequences in all three forward frames. TRANALIGN verified the retrieved CDS and validated the corresponding nucleotide sequences using the Swiss-Prot protein data. Validation was also repeated using the final gapped nucleotide alignment and the PFAM family alignment to ensure high quality of the sequence data.

Phylogenetic trees available from PFAM were utilized. These were calculated using an approximately maximum-likelihood neighbor joining approach based on 100 resamples using the FastTree algorithm (13). A computationally less expensive approach to prune those missing nucleotide sequences was adopted. The pruning algorithm removed missing sequences by collapsing the connecting nodes, if there was bifurcation, the branch lengths of the pruned sequences were added to the existing sequences.

Pruned trees and nucleotide sequences were then used to determine the ω profiles. The Bayes Empirical Bayes (1) ω MLEs at codon sites were calculated using the CODEML program (PAML ver. 4.4) (3) under the M2a Model (NSsites = 2). This parameter assumes one ratio for all the branches and allows for the detection of positive selection at codon sites. MLEs for ω were extracted from the ‘rst’ CODEML output file and used to compile the ω MLE profiles for each family. In addition, we also provide the analysis results under the M1a model (nearly neutral) for comparison.

## Results and discussion

**Figure 2.**
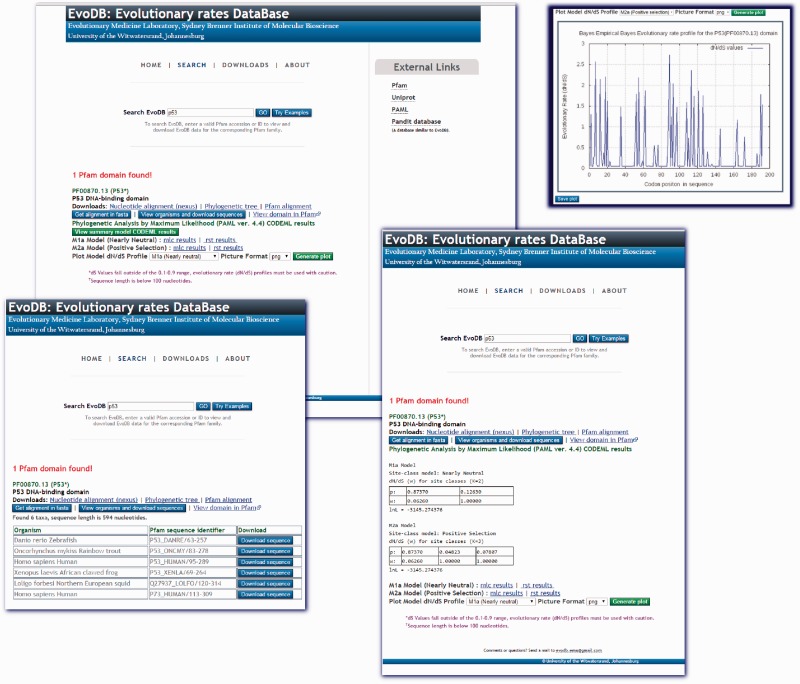
The EvoDB web interface allows for easy query and download of data. The database can be queried using PFAM-A domain identifiers and accession identifiers. The results shown here are for the tumor suppressor p53 domain. The CODEML ‘mlc’ and ‘rst’ analysis results for the M1a and M2ac models are provided and a summary of results is provided for viewing. Graphical plots of evolutionary rate profiles can also be viewed or downloaded in various picture file formats. EvoDB provides an interface for downloading the corresponding nucleotide sequences of PFAM protein domain families.

EvoDB is a flat file database of evolutionary rate profiles, associated gapped nucleotide alignment, phylogenetic trees and corresponding PFAM alignments for the PFAM-A seed alignments database. The database statistics are provided in Table 1. EvoDB contains a total of 501,375 nucleotide sequences, indicating that 176,757 (26%) could not be retrieved, this was mostly due to annotation errors, an increasing challenge which has not been addressed since the work of (8). Additionally, the corresponding phylogenetic trees, PFAM-A alignments and accession identifier data on all sequences including those that could not be retrieved are provided in the database. Evolutionary rates profiles were determined for 97.1% of PFAM-A entries under the M2a model. In addition to these profiles, CODEML analysis results for the M1a and M2a models are provided for comparison and hypothesis testing. Future versions of EvoDB will provide data for M0, M7 and M8 models. The efficacy of the model used to determine this evolutionary profile can be assessed by using the log-likelihood values or the Likelihood Ratio Test (LRT) (3) using the CODEML ‘mlc’ and ‘rst’ files provided. While we provide the evolutionary rate profiles (under M2a) for all the domains in EvoDB, the caveat is that calculation of d*N*/d*S* may be inappropriate for sequences that may have become highly diverged, say over millions of years or for closely related sequences. We suggest a criterion for total branch d*S* in the range of 0.1 and 0.9 found in the CODEML ‘mlc’ file, those domains not meeting this criterion may not be appropriate for d*N*/d*S* calculation. Users of the web interface are cautioned if a domain has a sequence length less than the 100 nucleotides or a total d*S* value outside the criterion. However, we provide this as a guideline and suggest caution and further interrogation when using d*N*/d*S* profiles from those domains that do not meet this criterion. On the other hand, the sequence data and trees are provided; therefore, different models can be run and assessed using the log-likelihood values or the LRT (3). The web interface for EvoDB was developed with PHP and JavaScript and can be queried by PFAM accession numbers or identifiers. Query results provide links to all the EvoDB data for the corresponding domain (Figure 2). The EvoDB database and release notes are available for download at http://www.bioinf.wits.ac.za/software/fire/evodb.

**Table 1.** Statistics of the EvoDB database representation for the PFAM-A seed alignments database

Sequence data	Numbers		Percentage	
	Pandit	EvoDB	Pandit	EvoDB
Evolutionary rate (ω MLE) profiles	—	13 277	—	97.1
Nucleotide sequence alignments	7738	13 512	56.6	98.83
Nucleotide sequences	174 760	501 375	25.8	74

The numbers of corresponding sequence data in Pandit (Pandit-Plus) have been provided for comparison. The percentage represents comparison of EvoDB coverage to the total numbers found in the PFAM-A seed alignments database.

EvoDB represents a valuable resource for phylogenetic studies, and can be used to test hypotheses in molecular evolution. It represents a tier of information untapped by current databases and will complement the arsenal of tools in phylogenetic studies.
